# Consistency of 3D femoral torsion measurement from MRI compared to CT gold standard

**DOI:** 10.1186/s12891-021-04633-7

**Published:** 2021-08-28

**Authors:** Thomas Vincent Häller, Pascal Schenk, Lukas Jud, Armando Hoch, Tobias Götschi, Patrick Oliver Zingg

**Affiliations:** 1 Department of Orthopedics, Balgrist University Hospital, University of Zurich, Forchstrasse 340, 8008 Zurich, Switzerland; 2 Computer Assisted Research and Development Group, Balgrist University Hospital, University of Zurich, Forchstrasse 340, 8008 Zurich, Switzerland

**Keywords:** 3D, Three-dimensional, Femoral torsion, Antetorsion, FAI, MRI

## Abstract

**Background:**

Several hip and knee pathologies are associated with aberrant femoral torsion. Diagnostic workup includes computed tomography (CT) and magnetic resonance imaging (MRI). For three-dimensional (3D) analysis of complex deformities it would be desirable to measure femoral torsion from MRI data to avoid ionizing radiation of CT in a young patient population. 3D measurement of femoral torsion from MRI has not yet been compared to measurements from CT images. We hypothesize that agreement will exist between MRI and CT 3D measurements of femoral torsion.

**Methods:**

CT and MRI data from 29 hips of 15 patients with routine diagnostic workup for suspected femoroacetabular impingement (FAI) were used to generate 3D bone models. 3D measurement of femoral torsion was performed by two independent readers using the method of Kim et al. which is validated for CT. Inter-modalitiy and inter-reader intraclass correlation coefficients (ICC) were calculated.

**Results:**

Between MRI and CT 3D measurements an ICC of 0.950 (0.898; 0.976) (reader 1) respectively 0.950 (0.897; 0.976) (Reader 2) was found. The ICC (95% CI) expressing the inter-reader reliability for both modalities was 0.945 (0.886; 0.973) for MRI and 0.957 (0.910; 0.979) for CT, respectively. Mean difference between CT and MRI measurement was 0.42° (MRI – CT, SD: 2.77°, *p* = 0.253).

**Conclusions:**

There was consistency between 3D measurements of femoral torsion between computer rendered MRI images compared to measurements with the “gold standard” of CT images. ICC for inter-modality and inter-reader consistency indicate excellent reliability. Accurate, reliable and reproducible 3D measurement of femoral torsion is possible from MRI images.

## Background

Femoral torsion was described as the angle between the femoral neck and the femoral condyles by Julius Wolff in 1868 [1, 2]. Several pathologies are associated with aberrant femoral torsion, such as slipped capital femoral epiphysis, developmental dysplasia of the hip and early-onset hip osteoarthritis [3–5]. There is an association between reduced femoral antetorsion and cam-type femoroacetabular impingement (FAI) [6–8].

Clinical quantification of femoral torsion is not reliable [6, 9, 10]. Initially standard radiographs such as the Dunn and modified Dunn view were used [1, 2, 11]. This has been replaced by more precise computer tomography (CT) and magnetic resonance imaging (MRI) measurements with various differences in measurement techniques for both of them [12, 13]. All these conventional methods that use cross sectional CT, MRI or ultrasound are two-dimensional (2D) imaging methods and encounter problems representing the complex, three-dimensional (3D) structure of the femur. To overcome the limitations of conventional 2D imaging methods, a 3D imaging method was developed by Kim et al. with greatly improved accuracy compared to conventional 2D imaging methods [14, 15]. These 3D measurements require CT imaging which has become the “gold standard” for quantification of 3D bone morphology in patients with structural hip disorders, such as FAI [16–20]. But using CT to characterize 3D hip morphology in the mostly young population of FAI patients is controversial due to potential harmful ionizing radiation exposure (about 4-5 mSv per CT) with a small lifetime attributable risk (0.034–0.177% for a 20-year-old) but a large relative risk (5–17 times) of cancer compared with radiographs alone [21, 22]. On the other hand MRI is useful to evaluate intra- and extra-articular soft tissue structures and the cartilage [23–28]. Thereby it provides essential predictors for the benefit of FAI surgery [29].

It would be preferable to measure the femoral torsion in 3D from MRI images without the need of additional CT imaging and consequently avoiding harmful ionizing radiation and save healthcare resources.

To understand complex deformities 3D measurements can be helpful. That is why they are desirable to get, best with as little as possible additional expenses. Correction of such complex deformities in particular may benefit from 3D analysis and 3D planning of the deformity correction to minimize errors which could lead to biomechanical alteration [30–32]. The required 3D bone models can be generated from CT or MRI data [33–37].

However, the proof that 3D femoral torsion measurements from MRI generated 3D models correspond with measurements from CT generated 3D models has not occurred yet. The aim of this paper was to investigate if 3D measurement of femoral torsion from MRI yielded comparable results as measurement from CT data. We hypothesized that agreement existed between reconstructed MRI and CT 3D measurements of femoral torsion.

## Methods

### Patient selection

The local ethical committee approved this study (BASEC Number 2012–02242) and all patients gave their informed written consent for their participation and the publication of this study.

We retrospectively analysed CT and MRI data of 29 hips from 15 patients (7 female, 8 male) who had routine workup for clinical symptoms suggestive for FAI between May and November 2019. The average age at the time of the scans was 32 years (range 21–47 years). Fourteen patients had an MRI and a CT scan of both sides. One patient had imaging only of the right side, which lead to a total of 29 hips (15 right hips, 14 left hips) with complete MRI and CT dataset.

### Imaging and segmentation

All CT scans were performed at our institution, using a 64-detector row CT scanner (Somatom Edge Plus, Siemens Healthcare, Erlangen, Germany), slice thickness was 1.0 mm. The protocol was as follows: Feet first, supine; central positioning of the pelvis, slightly internal rotation of the legs; image centered in the middle of the pelvis slightly above the iliac crest. The proximal femur was scanned from just above the iliac crest to the end of the lesser trochanter. The distal femur was scanned from the femoral condyles to the joint line.

MRI were also performed at our institution using a 3.0 Tesla MR scanner (Magnetom Skyra 3.0 T; Siemens Healthcare, Erlangen, Germany). The protocol was as follows: Coronal 3D T1-weighted VIBE-Dixon sequence (femur, bilateral): Slice thickness 1.5 mm, FOV 348x655mm, Echo time 5.7 ms, Repetition Time 2.5 ms, number of images 88.

CT and MRI scans were performed on the same day. No intraarticular contrast was given for CT scans nor for MRI.scans.

### 3D measurement method

**Fig. 1:**
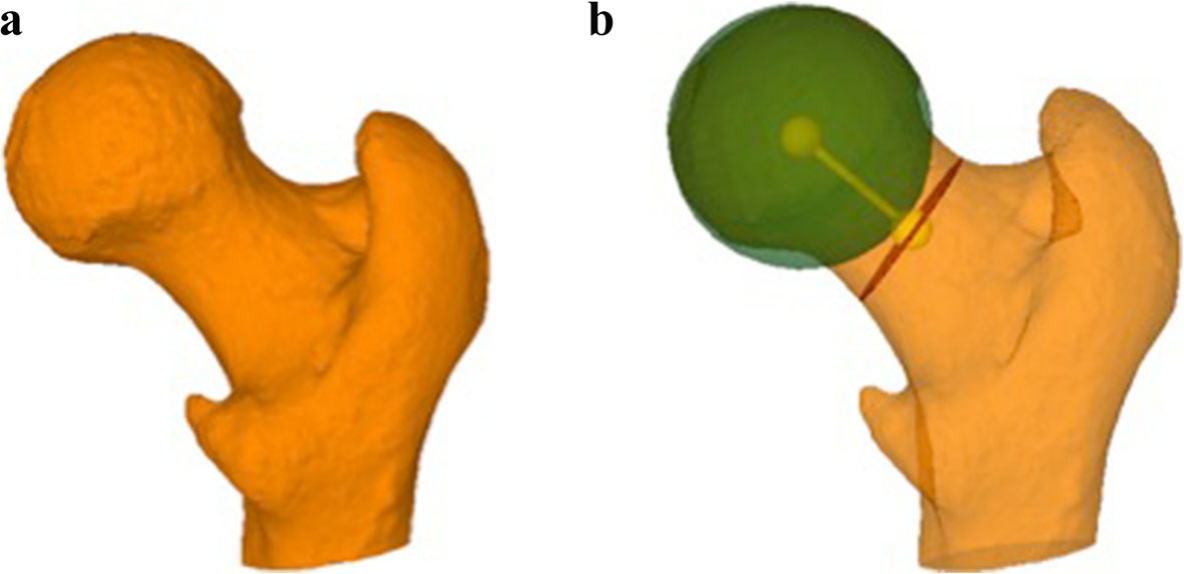
**a** 3D model of the proximal femur from CT data. **b** The same 3D model then 1a with the sphere fitted to the femoral head (green), the cross section at the narrowest point of the femoral neck (red) and the femoral neck axis (yellow), connecting the two centers. Both figures were created by the authors using the in-house developed surgical planning software CASPA (Balgrist CARD AG, Zurich, Switzerland)

**Fig. 2:**
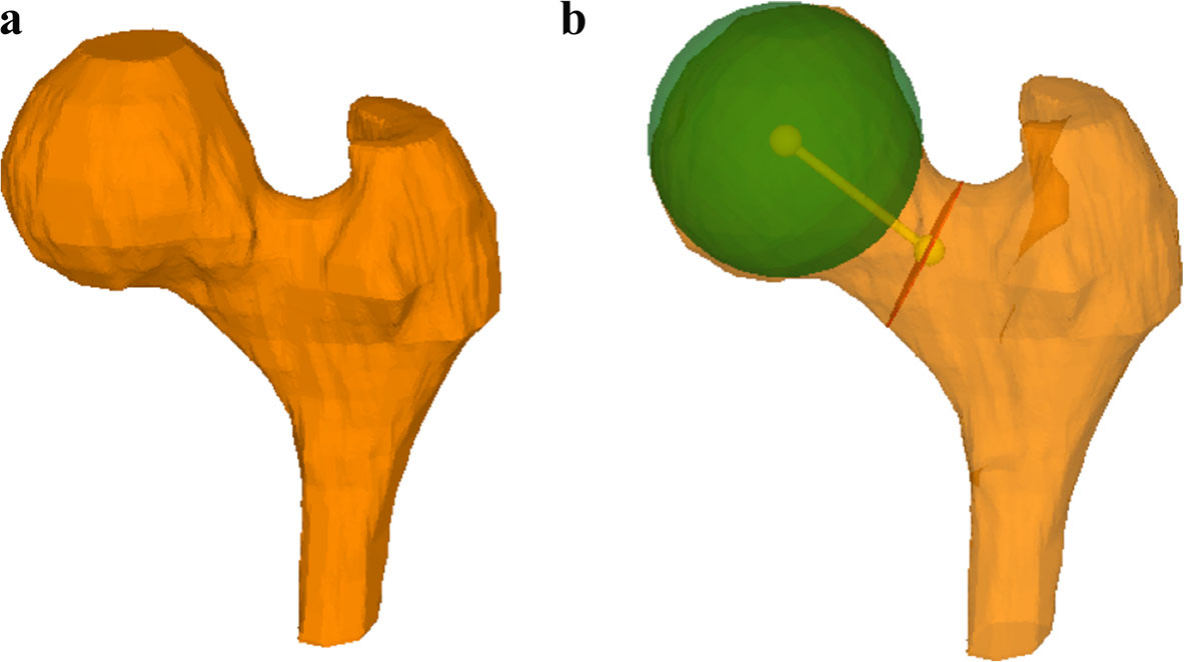
**a** 3D model of the proximal femur from MRI data. (Note the much less precise osseous morphology compared to the CT model shown in Fig. 1). **b** The same 3D model with the sphere fitted to the femoral head (green), the cross section at the narrowest point of the femoral neck (red) and the femoral neck axis (yellow), connecting the two centers. Both figures were created by the authors using the in-house developed surgical planning software CASPA (Balgrist CARD AG, Zurich, Switzerland)

3D bone models of all included femurs were generated from CT and MRI data using the global thresholding and region growing functionality of a standard segmentation software (Mimics Medical 19.0, Materialise NV, Leuven, Belgium). Segmentation of the MRI was performed using the T1-weighted VIBE-Dixon sequence. The bone models were imported into the in-house developed surgical planning software CASPA (Balgrist CARD AG, Zurich, Switzerland) for 3D measurement of the femoral torsion (Figs. 1a and b and 2a and b).

**Fig. 3:**
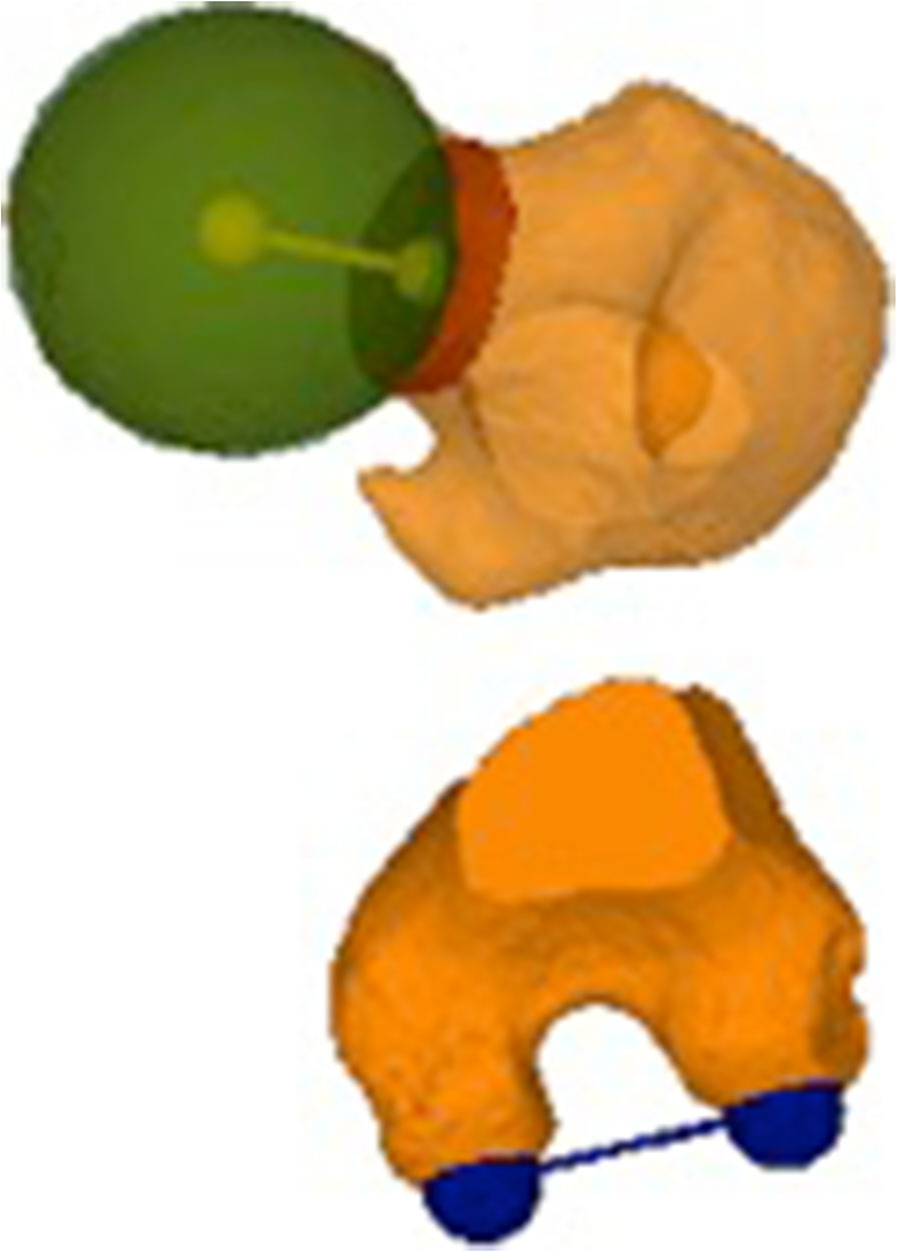
Femoral condylar axis (blue) and femoral neck axis (yellow) of a CT rendered image. The figure was created by the authors using the in-house developed surgical planning software CASPA (Balgrist CARD AG, Zurich, Switzerland)

3D femoral torsion measurements were performed subsequently using a method based on Kim et al. [15]. Thereby, femoral torsion is defined as the angle between the femoral neck axis and the tangent to the posterior condyles, both projected to a plane perpendicular to the anatomical axis (Fig. 3). The femoral neck axis is defined as the line connecting the center of the femoral head and the center of the cross-section at the narrowest point of the femoral neck. First, the center of the femoral head was determined by fitting a sphere to the femoral head, minimizing the distance to a user-selected region on the femoral head [38]. Second, a plane was manually fit to the narrowest diameter of the femoral neck, perpendicular to the estimated femoral neck axis. The center point of the intersection between this plane and the femoral bone was connected with the center of the femoral head, resulting in the femoral neck axis.

The tangent to the posterior condyles was determined by manually fitting a plane to the posterior side of the femur, in a way that the plane passed through the most posterior points of the femoral condyles and the most posterior point of the greater trochanter. The condylar tangent was then defined as a line between most posterior points of the femoral condyles.

Finally, the anatomical femoral axis was defined between the centre of an axial cross-section located in the middle of the tip of the lesser and the greater trochanter and the centre of an axial cross-section just above the femoral condyles. By using the three defined axes (i.e. femoral neck axis, femoral condylar axis, and anatomical femoral axis), the 3D femoral torsion was calculated in MATLAB (Version 2019a, The MathWorks Inc., Natick MA, USA). For inter-reader reliability all 3D femoral torsion measurements were performed by two independent readers (TH and LJ).

### Statistical analysis

Inter-reader and inter-modality reliability were assessed with intraclass correlation coefficients (ICC) based on a two-way random effects and a two-mixed effects model, respectively. Absolute agreement based on single measures was analyzed. These analyses were stratified by reader or by modality as applicable. The standard error of measurement was computed to yield an estimate of the expected error associated with a measurement. To test for a systematic difference in angle measurements between the modalities, a paired t-test was conducted. This test was applied on the pooled data from both readers. Intra-observer variability between CT and MRI for both readers were also assessed. Analysis was performed with SPSS (IBM SPSS Statistics for Windows, Version 26.0. Armonk, NY: IBM Corp.). *P*-values below 0.05 were considered statistically significant.

## Results

Individual 3D measurement values of both readers from CT and MRI data for all 29 hips are shown in Table 1. The range of the measured 3D femoral torsion was from  -16.4° to 28.2°.

**Table 1:** 3D torsional measurements from CT and MRI from reader 1 and reader 2. Values in °

Subject	CT	MRI	*CT*	*MRI*	CT	MRI	*CT*	*MRI*
	Reader 1	Reader 1	*Reader 2*	*Reader 2*	Reader 1	Reader 1	*Reader 2*	*Reader 2*
	RIGHT HIP				LEFT HIP			
1	14.5°	14.9°	16.4°	18.1°	17.5°	16.5°	19.4°	16. 2°
2	−9.4°	−0.6°	−5.3°	−4.1°	−4.7°	n.a.	−0.2°	n.a.
3	15.2°	14.1°	13.1°	14.2°	18.2°	18.8°	18.3°	21.4°
4	12.4°	8.9°	15.1°	11.4°	9.5°	13.0°	13.5°	16.6°
5	7.6°	10.9°	4.6°	9.5°	10.0°	12.5°	10.2°	8.3°
6	10.5°	11.2°	9.1°	6.5°	4.7°	7.3°	6.2°	7.5°
7	11.9°	8.6°	8.1°	5.5°	9.1°	10.4°	8.1°	11.6°
8	20.7°	18.6°	21.4°	19.3°	28.2°	27.4°	27.0°	24.2°
9	5.4°	7.6°	8.0°	8.2°	9.0°	6.8°	10.6°	11.4°
10	1.9°	0.2°	1.0°	2.8°	2.8°	3.2°	5.8°	6.1°
11	5.4°	3.3°	0.2°	0.8°	20.8°	20. 6°	18.3°	19.0°
12	−7.2°	−4.1°	−2.3°	−7.2°	−15.8°	−16.4°	−13.2°	− 16.0°
13	7.4°	7.4°	10.0°	8.5°	3.3°	3.1°	4.8°	6.2°
14	14.8°	15.8°	14.5°	13.0°	17.1°	13.8°	17.1°	18.2°
15	10.1°	14.4°	14.4°	17.6°	2.6°	7.3°	5.4°	12.2°

### Inter-modality reliability

**Fig. 4:**
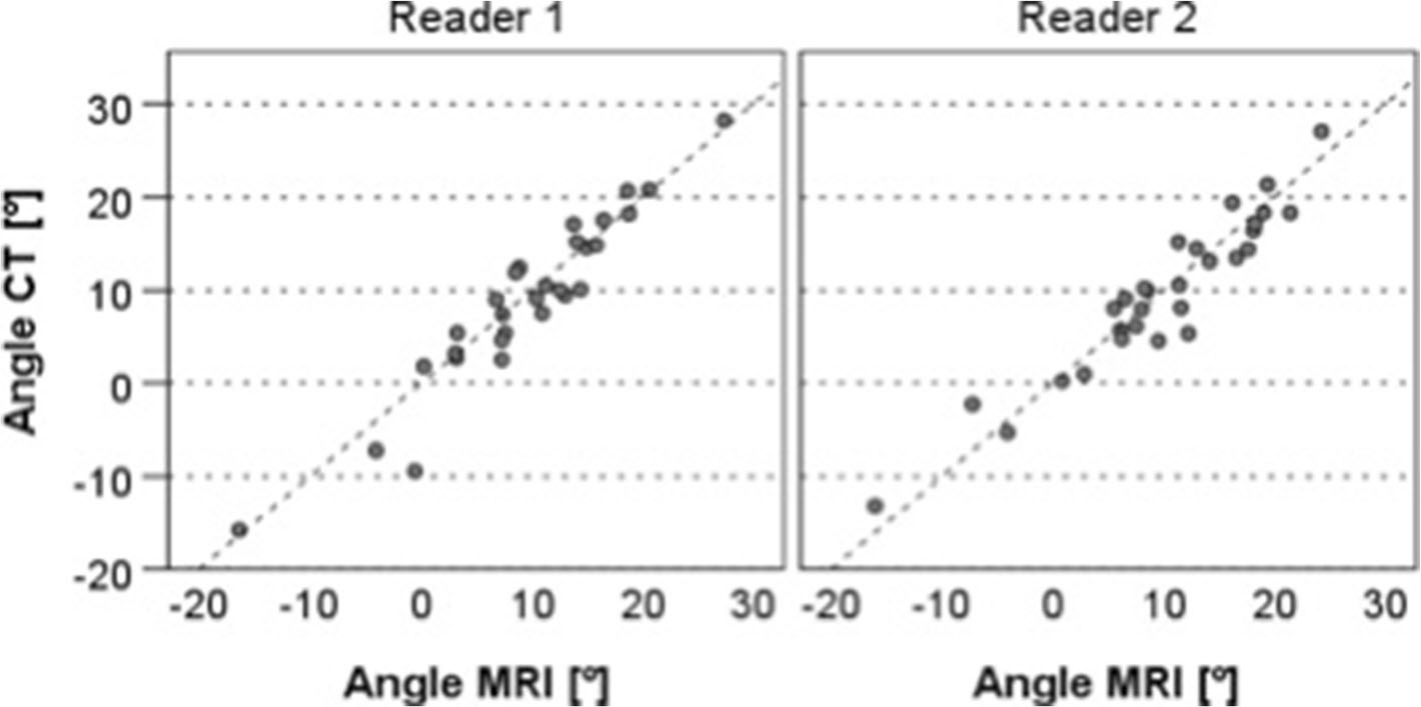
Comparison of the measurements based on the two imaging modalities assessed, for reader 1 and reader 2

The ICC (95% CI) expressing the inter-modality reliability for both assessed modalities was 0.950 (0.898; 0.976) (reader 1) and 0.950 (0.897; 0.976) (reader 2), respectively. The standard error of measurement (SEM) was 1.97° (reader 1) and 1.92° (reader 2), respectively (Fig. 4).

The comparison of angle measurements conducted with either modality yielded a mean difference of 0.42° (MRI – CT, SD: 2.77°, *p* = 0.253).

### Inter-reader reliability

**Fig. 5:**
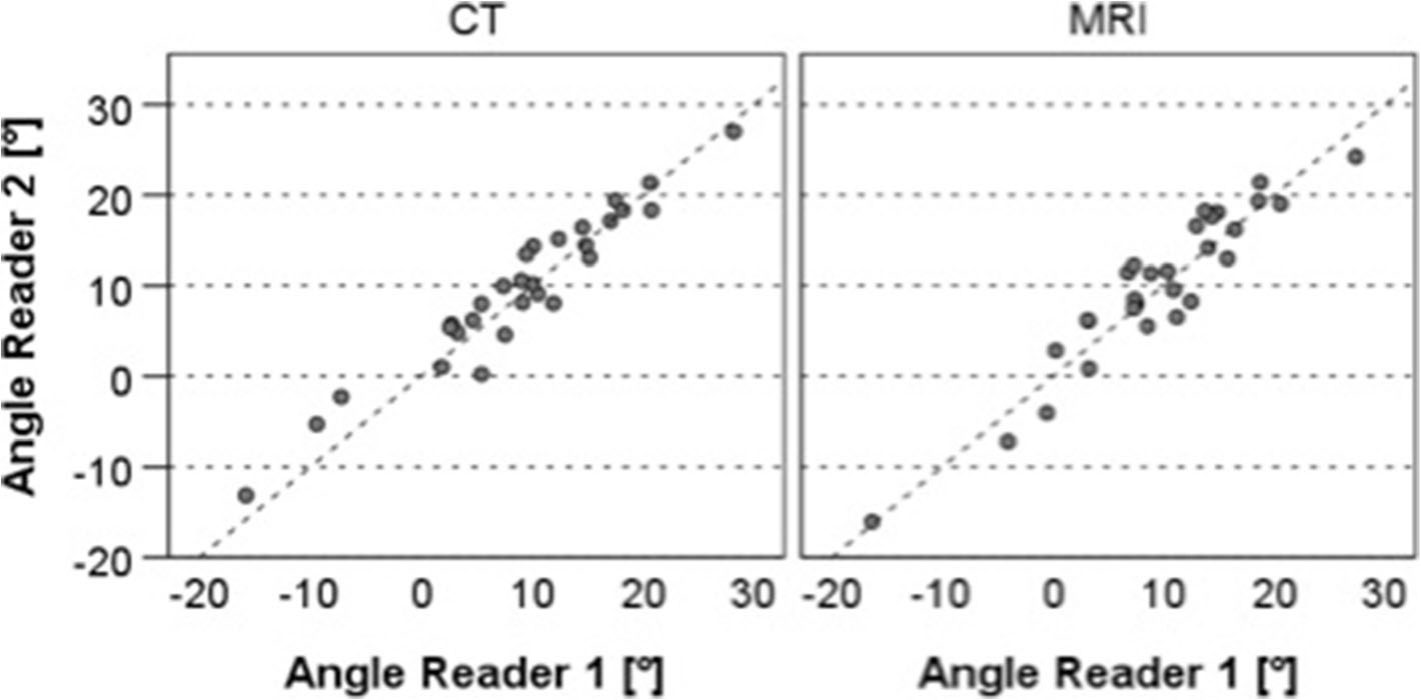
Comparison of the measurements performed by the two readers for both image modalities

The ICC (95% CI) expressing the inter-reader-reliability for both modalities was 0.945 (0.886; 0.973) for MRI and 0.957 (0.910; 0.979) for CT. The standard error of measurement (SEM) was 2.01° (MRI) and 1.83° (CT), respectively (Fig. 5).

### Intra-observer variability

The mean intra-observer variability between CT and MRI was 1.51 (reader 1) respectively 1.62 (reader 2).

## Discussion

The most important finding of the present study is that consistency exists between 3D measurements of femoral torsion from MRI and CT reconstructed bone models. The mean difference of the modalities was very low with 0.42° (MRI – CT, SD: 2.77°, *p* = 0.253). Our hypothesis was confirmed further by an ICC for the inter-modality reliability of > 0.9 expressing an excellent reliability [39]. Additionally, inter-reader reliability was estimated to be excellent indicating high reproducibility of the measurements. The SEM was 1.97° (reader 1) respectively 1.92° (reader 2). In our opinion the MRI can still provide valuable information with a measurement error of 2°.

Three dimensional femoral torsion measurements with computer rendered CT images using the 3D modeling method described by Kim et al. is the “gold standard” [15]. On computer rendered MRI data the anatomy may be depicted less precise than on CT data, since some landmarks (i.e. the lesser trochanter) are more difficult to identify (see Figs. 1 and 2), slice thickness of MRI rotational sequences is usually higher (i.e. 1 mm for CT vs. 1.5 mm for MRI in this study) and soft tissue is more difficult to distinguish from bone and cartilaginous tissue during the segmentation process. Surprisingly, these large differences do not seem to have a significant impact on the final result of the 3D torsional measurements. MRI is more time consuming than CT (about 91 seconds vs. 5 seconds) and so there is a potential for movement of the patient’s leg between scanning the proximal femur and the condylar region and thereby influencing the torsional angle. But still the chance of complete failure of the MRI is negliable. Anyhow, this is a hypothesis of the authors and could not be proved by this study. It is therefore crucial that the rotational sequences are scanned as quickly as possible in succession. The great advantage of MRI is that it can analyze cartilage and soft tissue disorders in addition to bone conditions whereas the CT is considered to be more precise for evaluating the bony anatomy. However, previous studies have shown that MRI can be used as a precise alternative to CT for evaluation of 3D osseous morphology in the shoulder and knee [33, 36]. Moreover, the identification and location of cam-type morphology was equivalent using 3D osseous reconstructions of the femur generated from 3 T MRI and CT scans [40]. However, none of these studies could show that 3D measurement of patients’ femoral torsion could be performed from MRI data instead of CT data. To our knowledge this is the first study that calculates 3D femoral torsion using MRI data comparing it to 3D femoral torsion measurements form CT data.

Our results indicate that 3D measurement of femoral torsion can be performed using MRI data, making the routinely performed CT imaging for measuring the 3D torsional angle questionable and, thus, giving a potential to reduce healthcare resources and harmful ionizing radiation.

We acknowledge limitations of our study. The performed measurement was not analyzed for subgroups of patients with specific torsional angles (i.e. normal, too high, too low torsion). Therefore we can only state that the two measurement methods are comparable within the range tested in our collective. The number of patients in this study would be too small to analyze subgroups with “extreme” torsional angles and the goal was primarily to evaluate the accuracy of the measurement from MRI data in general and should be considered as a preliminary study that serves as proof of concept.

Further MR image segmentation was performed by a person without prior academic training in the matter but he was carefully instructed to carry out the task. Individuals with different levels of training may yield varying results.

## Conclusions

Accurate 3D measurement of femoral torsion is possible from MRI images. It shows consistency compared to measurements with the “gold standard” of CT images in our collective of 29 hips.

## Data Availability

Anonymized source data can be obtained from the corresponding author on reasonable request.

## Abbreviations

2D: Two-dimensional
3D: Three-dimensional
CT: Computer Tomography
FAI: Femoroacetabular impingement
ICC: Intraclass correlation coefficients
MRI: Magnetic Resonance Imaging
